# Rapid evolution of virulence leading to host extinction under host-parasite coevolution

**DOI:** 10.1186/s12862-015-0407-0

**Published:** 2015-06-13

**Authors:** Charlotte Rafaluk, Markus Gildenhard, Andreas Mitschke, Arndt Telschow, Hinrich Schulenburg, Gerrit Joop

**Affiliations:** Evolutionary Ecology and Genetics, Zoological Institute, Christian-Albrechts-Universitaet zu Kiel, Am Botanischen Garten 1-9, Kiel, 24118 Germany; Department of Zoology, University of Oxford, The Tinburgen Building, South Parks Road, Oxford, OX1 3PS UK; Department of Vector Biology, Max-Planck-Institut für Infektionsbiologie, Campus Charité Mitte, Charitéplatz 1, Berlin, 10117 Germany; Institute for Phytopathology and Applied Zoology, University of Giessen, Heinrich-Buff-Ring 26-32, Gießen, D-35392 Germany; Westfälische Wilhelms-Universität Münster, Institut für Evolution und Biodiversität, Hüfferstr. 1, 48149 Münster, Germany

**Keywords:** Microsporidia, Tribolium, Paranosema, Experimental evolution, Transmission, Curse of the pharaoh

## Abstract

**Background:**

Host-parasite coevolution is predicted to result in changes in the virulence of the parasite in order to maximise its reproductive success and transmission potential, either via direct host-to-host transfer or through the environment. The majority of coevolution experiments, however, do not allow for environmental transmission or persistence of long lived parasite stages, in spite of the fact that these may be critical for the evolutionary success of spore forming parasites under natural conditions. We carried out a coevolution experiment using the red flour beetle, *Tribolium castaneum*, and its natural microsporidian parasite, *Paranosema whitei*. Beetles and their environment, inclusive of spores released into it, were transferred from generation to generation. We additionally took a modelling approach to further assess the importance of transmissive parasite stages on virulence evolution.

**Results:**

In all parasite treatments of the experiment, coevolution resulted in extinction of the host population, with a pronounced increase in virulence being seen. Our modelling approach highlighted the presence of environmental transmissive parasite stages as being critical to the trajectory of virulence evolution in this system.

**Conclusions:**

The extinction of host populations was unexpected, particularly as parasite virulence is often seen to decrease in host-parasite coevolution. This, in combination with the increase in virulence and results obtained from the model, suggest that the inclusion of transmissive parasite stages is important to improving our understanding of virulence evolution.

**Electronic supplementary material:**

The online version of this article (doi:10.1186/s12862-015-0407-0) contains supplementary material, which is available to authorized users.

## Background

During the course of host-parasite coevolution, reciprocal genetic changes are predicted to occur between the host and parasite [1]. A key parasite trait often expected to change as a consequence of these dynamics is virulence [2, 3], which we define as the reduction in host fitness caused by a parasite [4]. Optimal virulence is reached by an evolutionary compromise between reproducing too rapidly within the host and reproducing at a suboptimal lower rate. Too rapid reproduction may result in reduction of their level of transmission, whereas too low reproduction would risk not extracting the maximum amount of possible resources from the host [3, 5]. The outcome is likely to be highly dependent on the genetic diversity in the parasite population [6, 7] and the transmission mechanism of the parasite [8, 9]. Thus, in experimental evolution the methods and parameters, often chosen by the experimenter arbitrarily or for practical reasons, can play a pivotal role in the results obtained. For example, starting a coevolution experiment from a single parasite genotype might result in a less virulent parasite evolving in comparison to an experiment starting from a mixed stock, as competition pressure between differing genotypes would increase with the level of genetic diversity present [10]. Furthermore, taking parasite propagules at a very specific time point may cause the parasite to evolve the optimal virulence for transmission at that time point rather than for the system as a whole.

The prediction of a trade-off between transmission and virulence is based on the idea that an increase in transmission rate should come at a cost in terms of duration of infection [2, 11, 12]. Obligatory killing parasites are particularly interesting to consider in this respect as in their case transmission is itself dependent on death. Consequently, a reduction in virulence potentially comes at an extremely high cost, as transmission success is reduced to zero if the host survives [5, 13]. In spite of this prediction, several studies have in fact provided evidence of a trade-off in obligatory killing parasites. Jensen *et al.,* [3] showed an intermediate level of virulence to be optimal in the obligately killing parasite of *Daphnia magna*, *Pasteuria ramosa.* Furthermore, Bérénos *et al.,* [5] showed that following co-evolution between *Tribolium castaneum* and its microsporidian parasite *Paranosema whitei* virulence was reduced with the apparent benefit of increased spore production.

The majority of host-parasite co-evolution experiments using animal systems do not allow for transmissive stages of parasites to persist in the environment. Mechanisms of transmission involving prolonged environmental stages are, however, wide-spread for intracellular parasites in nature, including many of the horizontally transmitted microsporidia [8]. Additionally, the presence of these stages has been hypothesised and theoretically predicted to have a substantial impact on virulence evolution, especially when they are long lived [14]. Known as “the curse of the pharaoh hypothesis”, mathematically formalised by Bonhoeffer *et al*., [15], the cost of virulence is suggested to be reduced for long lived parasites as they are able to endure periods of low host density or even complete host absence [14, 15]. Furthermore, the presence of transmissive stages in the environment can increase the genetic diversity of the parasite population, potentially increasing competition between parasite genotypes, resulting in higher virulence due to reduced returns in terms of inclusive fitness [16].

Consequently, the inclusion of transmissive stages in evolution experiments may be critical for the effects of a trade-off, or lack thereof, to be realised. If spores are extracted from dead hosts at a specifically defined time point, as in most experimental evolution protocols e.g. [5, 17–20], then spore numbers should be maximised for this exact time point and the virulence-transmission trade-off should be adjusted accordingly. If, however, transmissive stages are allowed to persist in the environment, as realistic under most natural conditions, then selection may also favour higher virulence levels, as the earlier produced spores will not be lost from the experiment. We carried out a host-parasite coevolution experiment using the red flour beetle, *Tribolium castaneum* and its natural, obligately killing microsporidian parasite *Paranosema whitei* [21–23] in order to test the consequences of environmental spore persistence on the dynamics of the interaction*. P. whitei* infects early stage larvae and usually kills in the late larval or early pupal stage [22, 23]. Spores are released upon host death and can persist for extended periods of time without losing infectivity [21]. In our experiment, parasite stages were given the opportunity to enter and remain in the environment, providing potential benefits of increased parasite longevity, and host generations were allowed to partially overlap. We additionally took a modelling approach in order to further explore our results in the context of both the presence of environmental parasite stages in general and in the specific case of the curse of the pharaoh hypothesis. We show that in our system incorporating environmental parasite stages, virulence increases as a consequence of host-parasite coevolution.

## Methods

### Host

*Tribolium castaneum* is a tenebrionid beetle, which inhabits human grain stores, with the earliest recorded association with humans being in Egyptian times [24]. The genetically diverse *Tribolium castaneum* population Cro1, recently isolated from a natural site in Croatia [25], was used to start the evolution experiment. The line had undergone around 20 generations of adaptation to standard lab rearing conditions prior to use in the experiment.

In order to produce adults of a similar age with which to start the experiment, 20 oviposition jars (Bardenhewer, Kiel, Germany) were set up five weeks prior to the start date. Each jar contained 140 g of flour (Alnatura type 550) plus 5 % brewer’s yeast (Leiber, Bramsche, Germany). Approximately 200 adult beetles, of the strain Cro1, were transferred to each jar. The beetles were then left to oviposit for 14 days, following which they were removed. Offspring were left to develop for a further three weeks, at the end of which there were a large number of adult beetles. 12 random jars were sieved and the adults taken off then mixed and used to start the experiment. Throughout the experiment beetles were stored at 32 °C with 70 % humidity and in complete darkness.

### Parasite

Microsporidia are single celled eukaryotes that obligately parasitize cells of other eukaryotic organisms [26]. *Paranosema whitei,* formerly *Nosema whitei* [27, 28], is a specific microsporidian parasite of scarabid beetles, particularly *Tribolium* spp. [21]. In order to generate enough microsporidian material to start the evolution experiment, material from an infected beetle population was split evenly between 5 standard 50 ml tubes (Sarstedt, Nümbrecht, Germany), the 50 ml tubes filled up to the 15 ml line with a mixture of flour (Alnatura type 550) plus 5 % brewer’s yeast and the tubes vortexed thoroughly at multiple angles. 20 beetles from an outbred population generated from 4 different lab stock lines, GA2, 50, 51 and 61 which are of varied geographical origin, excluding the Cro1 line, were allowed to oviposit on the inoculated flour for 3 days, following which the flour was sieved and the adults removed. The tubes were subsequently sieved weekly and dead larvae collected until all larvae had died or become adults. This process was repeated twice on the same flour. The collected larvae were finally ground with a pestle and mortar and used to start the experiment. Spores were stored in the fridge at 4 °C, under which conditions they have previously been shown to remain infective for extended periods of time [21].

### Experimental set-up

The evolution experiment consisted of four treatments: three different environmental concentrations of *P. whitei,* representing substantially differing levels of beetle mortality over a single generation, and a control treatment consisting of standard flour with 5 % yeast mixture. The *P. whitei* concentrations used were 10^2^ (low), 10^3^ (intermediate) and 10^4^ (high) spores per g flour. Concentrations were chosen to be in the range of those used in previous experiments [5, 22, 23] and to show strong differences in survival with the ancestral population based on preliminary assays (Rafaluk, unpublished). Each treatment group consisted of seven replicate populations.

To start the experiment the spore concentration of the ground larvae mixture was counted, using a “Fast Read 102” disposable counting chamber (Biosigma, EU), and a portion diluted to 10^4^ in a mixture of flour (Alnatura type 405) containing 5 % yeast. This flour spore mixture was vigorously mixed in a large jar to produce an even distribution of spores. The lower concentrations were diluted following the same method, but with the relevant amount of the spore flour mixture from the next highest treatment. Each population was started with 17 g of the relevant flour or flour spore mixture in a 50 ml tube. 100 age synchronised adults of the line Cro1, synchronised as described above but with a slightly longer oviposition period of 7 days, were then added to each population and left for three weeks to oviposit and their offspring to become pupae.

Following this initial 3 week period all populations were sieved. 11 g of the flour from the previous generation was put back into the tube and 5 g of new flour as food, containing the starting concentration of ancestral spores, was also added. A cage, made from a standard 15 ml tube (Sarstedt, Nuembrecht, Germany) with two 4 cm long sections removed and replaced by 1 × 0.5 mm plastic mesh, was inserted into each 50 ml tube containing the mixture of new and old flour and spore mixture for each replicate population. The remaining beetles from the parental generation were placed into this cage and the cage sealed. The aim of the cage system was to produce semi-natural conditions with overlapping host generations present, whilst still allowing for host generations to be distinguished. In addition the cage allowed for contact and chemical communication between parents and offspring. To start the new generation 100 pupae were counted and added to the area of the large tube outside of the cage. If there were less than 100 pupae, the largest larvae were added to make the number up to 100, when possible. As populations approached extinction this became no longer possible and as many larvae and pupae as were available were added. After two weeks, at the point when the pupae had become fully matured adults, the cage and adults from the previous generation were removed. This procedure was then repeated every 4 weeks until population extinction. The setup of the evolution experiment is schematically represented in Additional file 1: Figure S1.

**Fig. 1:**
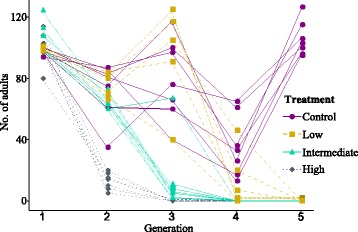
Absolute number of adults alive in each population at each generation of the evolution experiment. Treatments represent the starting concentration of *Paranosema whitei* used in the evolution experiment: low (10^2^ spores per gram flour), intermediate (10^3^ spores per gram flour) and high (10^4^ spores per gram flour)

At generation 2 for unavoidable practical reasons adults and larvae were separated 6 days early than in all previous and subsequent generations. However, due to the design of the experiment, specifically the presence of overlapping generations, this is unlikely to have had an effect on overall population size or infection dynamics, as adults were constantly present and producing susceptible larvae. This did, however, result in a dip in the number of adults present at the transfer across all treatments at generation two (Fig. 1).

### End concentration

As microsporidia spores are difficult to differentiate from flour grains via microscopy, the end spore concentration in the flour environment was determined by quantitative PCR (qPCR). A dilution series of spores, counted using the counting chamber method of spore quantification described above, was run in parallel for calibration. The 50 ml tube containing the final spore flour mixture was kept and transferred to the fridge for storage. Following extinction of all populations, 1 g of flour was sampled from each tube and total DNA extracted from the mixture using a standard Phenol-Chloroform extraction method. A subsample of the extracted DNA was then further purified (Machery-Nagel blood and Tissue DNA purification kit, Düren, Germany) for qPCR use and the quality and concentration checked using a Nanodrop**®** (ND-1000). qPCR was carried out using an Applied Biosystems 7300 qPCR machine in 15 μl reaction volumes with 20 ng of genomic DNA, pre-prepared qPCR mastermix with ROX (Eurogentec, Brussels, Belgium), the primers NW_16s_forw: 5’-GCT AGT CTT GGG GAC TTA GCC-3’ and NW_16s_rev: 5’-GGCCCT TAC CCT ACC TAC CA-3’ and a double labelled probe 5’-FAM-TAA GAC ATG GAC AGG CTG CA-6-BlackHoleQuencher1-3’, following the methods and conditions of Wegner *et al.* [29]. A dilution series of *P.whitei* DNA of known concentration was run in parallel for standardisation.

### Survival assay

For the survival assay dead larvae were taken from the experimental populations at the point of extinction, ground with a pestle and mortar and the spore concentration counted as described above. Collection of spores was only possible for the low and five of the intermediate treatments as the remaining intermediate and high populations went extinct before spore collection from dead larvae was possible. Spores were then diluted in flour to a concentration of 10^3^ spores per g, the initial intermediate concentration. The assay was carried out in small glass vials (40 × 13 mm) (Assistant, Sondheim/Rhoen, Germany) sealed with cotton wool filters (Eydam, Kiel), following the methods of Blaser and Schmid-Hempel [23]. 0.17 g of flour, where appropriate inoculated with the relevant spores, was weighed into each vial and a single second instar larva of the Cro1 line added. Larvae were generated by allowing age synchronised Cro1 adults to oviposit for 7 days as described above. Thirty vials, each containing an individual larva were set up for each isolate. The ancestral isolate and spore free flour were tested in parallel. Tubes were checked every other day to identify whether each individual was alive or dead, and this process continued until no new dead individuals were recorded at two successive time points.

### Spore load of individual dead larvae

Spore load was calculated for individuals infected with each microsporidian strain isolated from a population with a 10^2^ (low) starting concentration of microsporidia, hereafter referred to as “low isolate”, used in the survival assay for which a sufficient amount of material extracted from dead larvae taken from the experiment was available (five of the seven lines), as well as the ancestral parasite. Only the low line was used as the intermediate line did not show a consistent change in induced mortality in the survival assay. 10 adults of the Cro1 stock line were allowed to oviposit on 5 g flour containing 10^5^ spores per gram of the relevant isolate for 7 days, following which they were removed and discarded. Offspring were allowed to mature for a further 5 weeks, following which 3 dead larvae from each isolate were taken and ground individually in 1 ml water plus a small amount of Tween**®** 80 (Carl Roth, Karlsruhe, Germany) in a 2 ml tube (Sarstedt, Nuembrecht, Germany) using a 2000 Geno/Grinder ball mill homogeniser for 15 seconds at a speed of 500 rpm with 2 mm diameter steel balls. Spores present in the homogenised solution were subsequently counted using a “Fast Read 102” disposable counting chamber (Biosigma, EU). This method was used in order to most accurately obtain all the spores contained in a single larva.

### Statistical analysis of experimental data

All statistics were carried out in R version 2.15.1 (R core team 2013). Survival data were analysed first by pairwise ordinal-log-rank tests and subsequently using a series of pairwise binomial tests, both corrected for multiple testing using the false discovery rate (FDR) method, in order to compare each evolved line to the ancestral line at every time point. The pairwise binominal test method has been used previously by Henry *et al.,* [30] to assess homing probability in bees over time. The relationship between spore load and parasite induced mortality was analysed using a spearman’s rank test. All graphs were created using the R package ggplot2.

### The model

We took a modelling approach in order to determine whether the observed increase in virulence could potentially be explained by the environmental transmissive propagules of the parasite. We augmented the model of Anderson and May [31], which incorporates environmental parasite stages released upon host death, to include a larval and adult life-stage, in order to account for the structure of the host population. Additionally, following Bonhoeffer *et al.* [15], we incorporated the assumption that parasite density is not influenced by host uptake.

The basic model structure includes four variables. These are the numbers of pathogenic spores (*W*), susceptible larvae (*S*), infected larvae (*I*), and adults (*A*). Susceptible larvae are infected by the uptake of pathogenic spores from the environment at the rate β*W*S, where *β* is the transmission constant. Both infected and uninfected larvae develop at rate *m* into adults and both have a background mortality rate *d*. Further, infected larvae have an additional mortality *v* induced by the pathogen (virulence). Adults have a background mortality rate *u*. There is density dependent regulation of the adult population, which is modeled by carrying capacity *C*. Note that the infection status does not affect adult mortality. Spores are released to the environment when infected larvae die. This happens with the rate *z*, where *z* describes the presence or absence of a trade-off between virulence and spore production (see below). Spores decay in the environment at the rate *k*. These model assumptions yield the following system of ordinary differential equations.

1 $$ \frac{dA}{dt}=m\left(S+I\right)\left(1-\frac{A}{C}\right)-uA, $$

2 $$ \frac{dS}{dt}=-\beta SW+lA-\left(d+m\right)S, $$

3 $$ \frac{dI}{dt}=\beta WS-\left(v+d+m\right)I, $$

4 $$ \frac{dW}{dt}=zI- kW. $$

We analysed two versions of model (1)-(4). First, we considered a scenario where spores are shed into the environment upon host death, which makes spore production proportional to virulence. Following Anderson and May [31], we assumed that spore secretion increases linearly with virulence. This is described by the spore production rate z = *B* (*v + d*), where *B* is a constant that represents the infection load of a single infected individual. Secondly, we followed Boenhoeffer *et al*. [15] and assumed a case where spore production is a saturating function of virulence. We consider this a virulence spore production trade-off as it implies that an increase in virulence would increase the rate at which spores are secreted, but result in a reduced number of spores shed upon host death [32]. This is described by $z=\left(\frac{B}{v+x}\right)\left(v+d\right)$ where *x* is a trade-off constant.

The evolutionary dynamics were investigated using an extended model including two pathogen genotypes. Parameters were chosen to mimic the experimental conditions (see Additional file 1: Table S4). An invasion analysis of mutant genotypes was conducted to analyze evolution of virulence. Details of the extended model and how simulations were conducted can be found in the supplementary information.

## Results and discussion

All populations experimentally evolving with *P. whitei* went extinct between transfers 2 and 5 of the evolution experiment. By the third transfer there were no adults present in any population in the high treatment to start the next generation, by the fourth in the intermediate treatment and by the fifth in the low treatment (Fig. 1).

In order to establish whether an accumulation of parasite particles in the environment may have played a role, the end concentration of spores in the environment was measured by qPCR. Interestingly, regardless of the starting concentration of the population, or of the time until extinction, qPCR showed that the end concentrations of spores in the flour were all in the range of 10^6^ spores per g flour and did not differ significantly from one another (ANOVA: *F* = 0.42, *d.f*. = 2,15, p = 0.6) (Additional file 1: Table S1.). This is consistent with the findings of Milner [22], who showed 10^6^ to be a critical dose of *P. whitei*, above which the majority of individuals die as larvae. Our results therefore indicate that spore accumulation in the environment may have contributed to host extinction.

**Fig. 2:**
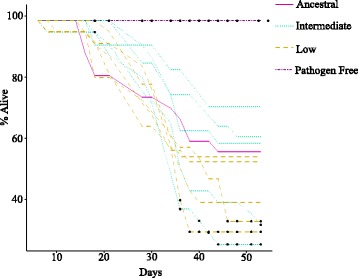
Percentage of individuals alive for each treatment at each time point. Percentage calculated in relation to the number of individuals recovered at the first time point. Filled black circles indicate time points at which each evolved line differed significantly from the ancestral line (pairwise binomial tests with fdr p value correction)

In spite of the observed increase in spore concentration it is possible that this alone was not responsible for host extinction and that an increase in virulence also contributed. To test this we carried out a survival assay. Spores from dead larvae were collected from five low and five intermediate treatments for use in the assay. All spore isolates from the initial low treatment populations showed higher mortality than the ancestral isolate. In the case of the isolates from the intermediate treatment populations the result was more varied with some showing increased mortality in comparison to the ancestral isolate and some showing a slightly lower mortality than the ancestral isolate (Fig. 2). In spite of the consistency in survival trends among the low treatment pairwise ordinal-log-rank test showed no significant difference between isolates (Additional file 1: Table S2). We hypothesised that this could be due to the fact that the survival curves diverged mainly toward the latter time points of the survival assay (Fig. 2). To test this we carried out pairwise binomial tests for each time point which indeed showed that towards the end of the assay the 4 most virulent isolates differed significantly in virulence from the ancestral population, with no lines being significantly less virulent than the ancestral line (Fig. 2; Additional file 1: Table S3). The fact that the most virulent isolates all come from the treatment which evolved the longest time before extinction, indicates that selection in our system increases virulence.

Virulence evolved extremely quickly in our system, with significant increases occurring within 5 host generations. Although this is seemingly few host generations, a single *P. whitei* infection can result in the range of 28 × 10^6^ spores being produced in a single host [23]. Consequently, this time period represents a potentially huge number of parasite generations providing plenty of scope for mutation and evolutionary change. Additionally, as our experiment was started from a parasite isolate rather than a single clone, there is likely to have been genetic diversity in the starting population allowing for rapid evolution [33].

There are several mutually non-exclusive reasons that could explain why only some of the intermediate treatment populations evolved higher virulence. One is that the opportunity for parasite evolution was constrained because the host killing rate, caused by higher starting concentration, was already at the upper limit for this experimental setting. As a consequence of the higher starting concentration, infection probability would have been higher for all genotypes, irrespective of their virulence. This could have resulted in semi-random infection of beetles by the various spore genotypes, thus leading to transmission of more or less all genotypes, precluding spread of the more virulent ones. A second potential explanatory factor is that, in the low population, any allele with heightened reproductive success would instantly contribute to a higher proportion of the total environmental spore composition than in the intermediate concentrate, making its spread more efficient and rapid.

The increase in virulence observed here is in contrast to the findings of other evolution experiments, including those using the same host-parasite system, where a significant decrease in virulence was seen to occur for exactly the same parasite *P. whitei* [5, 20]. However, our and the previous experiments aimed at approaching slightly different questions and therefore differed in central aspects of the methodology, potentially explaining this contrast. A major difference was the management of the parasite including the way in which the parasites were transferred from one generation to the next and the control of parasite concentration. In the experiments of Bérénos *et al.* [5, 20] transmission of spores to the next generation was based on collecting spores purely from dead larvae, with any spores that were released into the environment being discarded. In their case dead larvae were collected within a time window of 35–50 days post egg laying, whereas in our experiment dead larvae were removed after 28 days, meaning that larvae releasing spores way before the time window used by Bérénos *et al.* [5, 20] would have had a selective advantage. Furthermore, as spore concentration in the environment was carefully controlled by Bérénos *et al.* [5, 20], the level of competition for infection between spore genotypes may have been reduced. This difference in the methodology regarding transmission could potentially explain the discrepancies between our results and those of Bérénos *et al.* [5, 20].

**Fig. 3:**
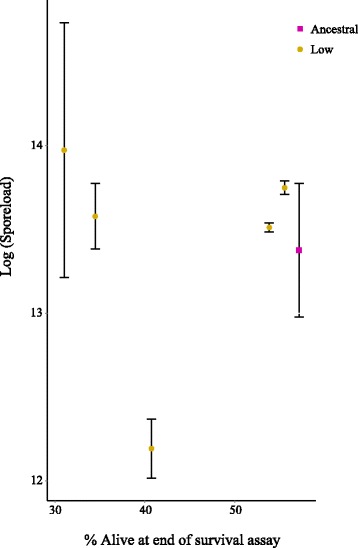
Correlation between percentage of individuals alive at the end of the survival assay and average spore load of individual larvae [count data] from 3 individuals from each line. Error bars represent standard error of the mean

In the experiments of Bérénos *et al.* [5, 20] a trade-off was observed between spore load of dead larvae, in the case of obligate killers a proxy for transmission, and virulence, which was the most parsimonious explanation for the observed decrease in virulence [5]. We, however, found no correlation between the level of virulence of each of the lines and the average spore load per larvae (Spearman’s rank test: S = 46, p = 0.56) (Fig. 3), meaning that in our case an increase in virulence did not appear to come at a cost of decreased transmission potential.

The lack of virulence/ transmission trade-off we see here, although consistent with some theoretical predictions [34], is inconsistent with previous empirical evidence that spore production/ virulence trade-offs do occur in obligately killing parasites [3, 5]. In contrast to previous studies with *P. whitei*, we did not observe obvious gigantism occurring in infected larvae [23], possibly resulting in reduced potential for spore production in our starting parasite population.

In order to further investigate the role of transmissive stages in the evolution of virulence we created a modified version of the “curse of the pharaoh” model [15] to fit the *T. castaneum*-*P. whitei* system. This approach allowed us to further explore the question of whether transmissive stages of the parasite being allowed to persist in the environment plays a role in the evolutionary dynamics that we would expect to see in this system.

A mathematical simulation of the survival assay we carried out produced similar mortality rates at differing levels of virulence to those seen with the evolved strains from the evolution experiment (Additional file 1: Figure S3). This suggests that increased virulence levels could potentially account for the increased mortality seen in the evolved lines. Additionally, in the model low host density, which we consider equivalent to host extinction due to the vulnerability of small populations to stochastic events, can only be achieved with increased virulence, with higher spore concentrations alone not leading to host extinction. This provides support to our hypothesis that increased virulence, rather than concentration alone, played a role in population extinction (Additional file 1: Figure S4).

**Fig. 4:**
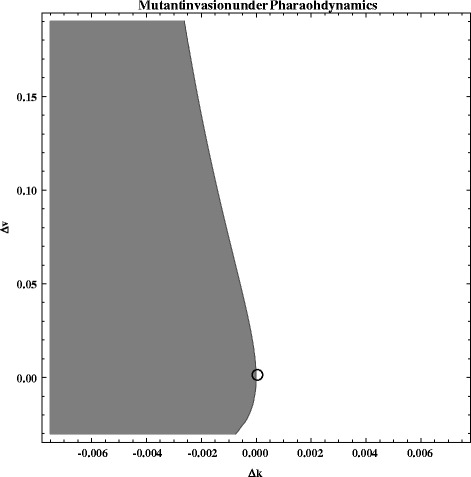
‘Pharaoh Dynamics’, a reduction of mortality may enable mutant invasion under the assumption that spore production is a saturating function of virulence: $z=\left(\frac{B}{v+x}\right)\left(v+d\right)$. The y-axis represents the difference (Δk = k2-k1) of mutant to the resident propagule mortality. The x-axis represents the difference (Δv = v2-v1) of the mutant virulence to the resident genotypes virulence. The grey area represents the cases where mutant invasion fitness *r > 0* and mutants can invade, as opposed to the white area, where r < 0 and mutants go to extinction. The point denotes the resident genotype, at which virulence mutants can only invade if virulence mutations are accompanied with a reduced spore mortality. The following parameter values were used in this simulation: *β* = 0.00083; m = 0.03; *u* = 0.07; *C* = 100; *l* = 0.33; *d* = 0.03; *k1* = 0.0075; *v1* = 0.03; *x* = 0.15; B = 750 000

Evolutionary invasion analysis [35] carried out with the model produced two possible explanations for host extinction. The first is that both reduced spore mortality and virulence evolved in parallel, reducing the cost of increased virulence as longer living spores are able to survive periods of temporary host absence or low population size under these circumstances, consistent with results based on the original model [15]. Under these circumstances high virulence in environmentally transmitted parasites should evolve if invasive mutants have higher survival outside of the host. If a mutation in virulence is accompanied by a reduction in spore mortality, these mutants will even invade systems in which a virulence mutation alone could not have invaded (Fig. 4).

**Fig. 5:**
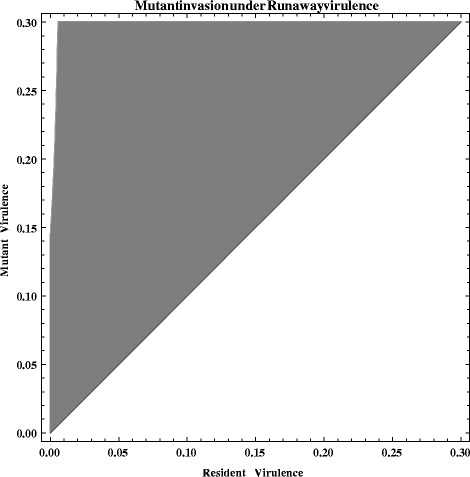
PIP ‘Runaway virulence’, pairwise invasion plot of virulence mutants with differing resident alleles in a case where spore production is proportional to virulence: *z* = *B*(*v* + *d*). The Grey area represents values of invader fitness r > 0. This fitness becomes positive for all mutants with higher virulence than the resident virulence. The same parameters were used as in Fig. 4: *β* = 0.00083; m = 0.03; *u* = 0.07; *C* = 100; *l* = 0.33; *d* = 0.03; *k1* = 0.0075; k2 = 0.0075; *x* = 0.15; B = 750 000

Interestingly however, when the trade-off between virulence and spore load was considered to be low or non-existent in the model, runaway virulence was predicted to evolve. Evolutionary invasion analysis showed that virulence mutants could invade a population of resident parasites at equilibrium if their reproductive factor was > 0 (Fig. 5). Given our finding that in our evolved material there did not appear to be a trade-off between virulence and spore load (Fig. 3) this would provide a more simple explanation than decreased spore mortality being the driving factor in the observed evolution of virulence.

Of the possible explanations for the increase in virulence observed here, we conclude that the hypothesis of a lacking trade-off between virulence and spore production is the most likely driver of the evolved increase in virulence. Despite the general presence of this trade-off being somewhat controversial [36–38], most published studies showing a change in virulence demonstrate that trade-offs of some kind provide a limitation on virulence evolution. Even in bacteria-bacteriophage systems where transmissive stages have been included, decreases in virulence over evolutionary time have been recorded. For example, Heineman and Brown [39] observed in an *Escherichia coli*- phage system that increased phage longevity came at a cost to reproductive rate, a proxy for virulence. In this system however, although phages were allowed to burst kill and persist in the environment, the persistence phase in harsh conditions was a separate experimental step from the replication in *E. coli*, potentially eliminating the possibility for alternative strategies to evolve and compete [39]. Examples of increased virulence do also exist, for example, Little *et al.*, [40] demonstrated in the *D. magna* – *P. ramosa* system that *P. ramosa*, a burst killer, increased in virulence upon adaptation to a specific host genotype without transmissive stages being present, even though King *et al.* [41] have demonstrated empirically that long lived stage are formed. This was, however, a serial passage experiment, where the host could not evolve in response and it has been shown empirically in the same system that an optimal level of virulence exists [3]. Our study is a rare example alluding to its potential presence of apparent run-away virulence in an empirical system.

It is important to note that in our experiment the parasite is not extinct, only the host, when considering the results of this experiment in relation to how virulence may evolve in nature. The ecology of the parasite is critical to its evolutionary success. If host migration would have been present in our experimental design, the parasite would not have been at a disadvantage at all as a result of making the contemporary host population extinct. As the spores are long lasting, even if a new susceptible host migrated into the environment a year in the future the parasite would be able to still infect it [21, 23]. Invasion by new host individuals or even a population is likely the case in nature and may explain why an obligately killing parasite was able to evolve in this system in the first place. Resultantly, the parasite would not necessarily lose any fitness advantage under natural conditions by displaying this trait [15, 42].

## Conclusions

When considering the evolutionary trajectories of traits under host-parasite co-evolution many ecological factors have to be considered and slight changes in these may have dramatic effects on the outcome. Our findings indicate that the presence of transmissive stages of a spore forming parasite may have an important influence on the outcome of evolutionary dynamics. We have also demonstrated that even in experiments with the same host-parasite system, differences in methodology and handling can lead to opposite results. All in all our results highlight the need to carefully consider all aspects of an organism’s ecology when studying virulence evolution. Furthermore, our model together with the empirical results highlight that the consideration of transmissive parasite stages clearly deserve more attention in empirical work on host-parasite co-evolution.

## Availability of supporting data

The data sets supporting the results of this article are included within the article and its additional files.

## Additional file

Additional file 1: Table S1. Start and end *P. whitei* concentrations of evolved populations. **Table S2.** p values of pairwise ordinal-log-rank tests comparing survival curves among a tested *P. whitei* isolates. Multiple testing was accounted for using the “fdr” correction. **Table S3.** Results of binomial tests comparing proportions of alive and dead beetles following exposure to each of the evolved P. whitei isolates in comparision to the ancestral isolate. In all cases comparison is to the proportion of indiviuals alive in the ancestral treatment on eac day. **Table S4.** Parameter values included in the model. These parameters were used to run simulations for all figures. **Figure S1.** Diagram of experimental set up of the evolution experiment. **Figure S2.** Schematic representation of the epidemiological model. **Figure S3**. Here we show how we estimated virulence levels from the survival assay. **Figure S4**. Here we show stable equilibrium points (y-axis) calculated from the model A, with just one genotype, over likely levels of virulence under experimental conditions (ranging between 0.01 and 0.09, see Additional file 1: Figure S3).

## References

[1] Hamilton WD (1980). Sex versus Non-Sex versus parasite. Oikos.

[2] Anderson RM, May RM (1982). Coevolution of hosts and parasites. Parasitology.

[3] Jensen KH, Little T, Skorping A, Ebert D. Empirical support for optimal virulence in a castrating parasite. PLoS Biol. 2006;4:e197. http://journals.plos.org/plosbiology/article?id=10.1371/journal.pbio.0040197. 10.1371/journal.pbio.0040197 PMC1470460 16719563

[4] Read AF (1994). The evolution of virulence. Trends Microbiol.

[5] Bérénos C, Schmid-Hempel P, Mathias Wegner K (2009). Evolution of host resistance and trade-offs between virulence and transmission potential in an obligately killing parasite. J Evol Biol.

[6] May RM, Anderson RM (1983). Epidemiology and genetics in the coevolution of parasites and hosts. Proceedings of the royal society of London. Series B Biol Sci.

[7] Barrett LG, Bell T, Dwyer G, Bergelson J (2011). Cheating, trade-offs and the evolution of aggressiveness in a natural pathogen population. Ecol Lett.

[8] Dunn AM, Smith JE (2001). Microsporidian life cycles and diversity: the relationship between virulence and transmission. Microbes Infect.

[9] Kisdi É, Boldin B (2013). A construction method to study the role of incidence in the adaptive dynamics of pathogens with direct and environmental transmission. J Math Biol.

[10] Read AF, Taylor LH (2001). The ecology of genetically diverse infections. Science.

[11] Bremermann HJ, Pickering J (1983). A game-theoretical model of parasite virulence. J Theor Biol.

[12] Massad E (1987). Transmission rates and the evolution of pathogenicity. Evolution.

[13] Ebert D, Weisser WW (1997). Optimal killing for obligate killers: the evolution of life histories and virulence of semelparous parasites. Proc Biol Sci.

[14] Ewald PW (1987). Transmission modes and evolution of the parasitism-mutualism continuuma. Ann N Y Acad Sci.

[15] Bonhoeffer S, Lenski RE, Ebert D (1996). The curse of the pharaoh: the evolution of virulence in pathogens with long living propagules. Proceed Biol Sci.

[16] Frank SA (1996). Models of parasite virulence. Q Rev Biol.

[17] Kolodny-Hirsch DM, Van Beek NAM (1997). Selection of a morphological variant of autographa californica nuclear polyhedrosis virus with increased virulence following serial passage in plutella xylostella. J Invertebr Pathol.

[18] Raymond B, Ellis RJ, Bonsall MB (2009). Moderation of pathogen-induced mortality: the role of density in Bacillus thuringiensis virulence. Biol Lett.

[19] Schulte RD, Makus C, Hasert B, Michiels NK, Schulenburg H (2010). Multiple reciprocal adaptations and rapid genetic change upon experimental coevolution of an animal host and its microbial parasite. PNAS.

[20] Bérénos C, Schmid-Hempel P, Wegner KM (2011). Experimental coevolution leads to a decrease in parasite-induced host mortality. J Evol Biol.

[21] Milner RJ (1972). *Nosema whitei*, a microsporidan pathogen of some species of *Tribolium*: I. Morphology, life cycle, and generation time. J Invertebr Pathol.

[22] Milner RJ (1972). *Nosema whitei*, a microsporidan pathogen of some species of Tribolium: III. Effect on *T. castaneum*. J Invertebr Pathol.

[23] Blaser M, Schmid-Hempel P (2005). Determinants of virulence for the parasite Nosema whitei in its host Tribolium castaneum. J Invertebr Pathol.

[24] Sokoloff, A. The biology of *Tribolium*. Clarendon Press. Oxford, UK, 1972.

[25] Milutinović B, Stolpe C, Peuβ R, Armitage SAO, Kurtz J (2013). The Red flour beetle as a model for bacterial oral infections. PLoS One.

[26] Keeling PJ, Fast NM (2002). MICROSPORIDIA: biology and evolution of highly reduced intracellular parasites. Annu Rev Microbiol.

[27] Sokolova YY, Dolgikh VV, Morzhina EV, Nassonova ES, Issi IV, Terry RS, et al. Establishment of the new genus *Paranosema* based on the ultrastructure and molecular phylogeny of the type species *Paranosema grylli* Gen. Nov., Comb. Nov. (Sokolova, Selezniov, Dolgikh, Issi 1994), from the cricket *Gryllus bimaculatus* Deg. J Invertebr Pathol. 2003;84:159–72. doi:10.1016/j.jip.2003.10.004. 10.1016/j.jip.2003.10.004 14726239

[28] Sokolova YY, Issi IV, Morzhina EV, Tokarev YS, Vossbrinck CR (2005). Ultrastructural analysis supports transferring Nosema whitei Weiser 1953 to the genus Paranosema and creation a new combination. Paranosema whitei. J Invertebr Pathol.

[29] Wegner KM, Berenos C, Schmid-Hempel P (2008). Nonadditive genetic components in resistance of the Red flour beetle *tribolium castanaeum* against parasite infection. Evolution.

[30] Henry M, Béguin M, Requier F, Rollin O, Odoux J-F, Aupinel P, Aptel J, Tchamitchian S, Decourtye A (2012). A common pesticide decreases foraging success and survival in honey bees. Science.

[31] Anderson RM, May RM (1981). The population dynamics of microparasites and their invertebrate hosts. Philosophical transactions of the royal society of London. Series B. Biol Sci.

[32] Garland T (2014). Trade-offs. Curr Biol.

[33] Nei M, Maruyama T, Chakraborty R (1975). The bottleneck effect and genetic variability in populations. Evolution.

[34] Gandon S, Hochberg ME, Holt RD, Day T (2013). What limits the evolutionary emergence of pathogens?. Phil Transact Royal Soc Biol Sci.

[35] Otto, S. P. & Day, T. A Biologist’s Guide to Mathematical Modeling in Ecology and Evolution. Princeton University Press. Princeton, New Jersey, USA, 2007.

[36] Ebert D, Bull JJ (2003). Challenging the trade-off model for the evolution of virulence: is virulence management feasible?. Trends Microbiol.

[37] Alizon S, Hurford A, Mideo N, Van Baalen M (2009). Virulence evolution and the trade-off hypothesis: history, current state of affairs and the future. J Evol Biol.

[38] Froissart R, Doumayrou J, Vuillaume F, Alizon S, Michalakis Y (2010). The virulence–transmission trade-off in vector-borne plant viruses: a review of (non-)existing studies. Philos Trans R Soc Lond B Biol Sci.

[39] Heineman RH, Brown SP (2012). Experimental Evolution of a Bacteriophage Virus Reveals the Trajectory of Adaptation across a Fecundity/Longevity Trade-Off. PLoS ONE.

[40] Little TJ, Watt K, Ebert D (2006). Parasite-host specificity: experimental studies on the basis of parasite adaptation. Evolution.

[41] King KC, Auld SKJR, Wilson PJ, James J, Little TJ (2013). The bacterial parasite Pasteuria ramosa is not killed if it fails to infect: implications for coevolution. Ecol Evol.

[42] Gandon S (1998). The curse of the pharaoh hypothesis. Proc Biol Sci.

