# Supplementary material for: Rapid evolution of virulence leading to host extinction under host-parasite coevolution
Source: BMC Evol Biol. 2015 Jun 13;15:112. doi: 10.1186/s12862-015-0407-0 (PMC4464865; doi:10.1186/s12862-015-0407-0)
Supplement: Additional file 1: Table S1. — Start and end P. whitei concentrations of evolved populations. Table S2. p values of pairwise ordinal-log-rank tests comparing survival curves among a tested P. whitei isolates. Multiple testing was accounted for using the “fdr” correction. Table S3. Results of binomial tests comparing proportions of alive and dead beetles following exposure to each of the evolved P. whitei isolates in comparision to the ancestral isolate. In all cases comparison is to the proportion of indiviuals alive in the ancestral treatment on eac day. Table S4. Parameter values included in the model. These parameters were used to run simulations for all figures. Figure S1. Diagram of experimental set up of the evolution experiment. Figure S2. Schematic representation of the epidemiological model. Figure S3. Here we show how we estimated virulence levels from the survival assay. Figure S4. Here we show stable equilibrium points (y-axis) calculated from the model A, with just one genotype, over likely levels of virulence under experimental conditions (ranging between 0.01 and 0.09, see Additional file 1: Figure S3). [file 12862_2015_407_MOESM1_ESM.docx]

**S.1.** *Model Description*

The model detailed below aimed to investigate the effect of transmissive parasite stages on virulence evolution in the *Tribolium castaneum* – *Paranosema whitei* system (see Table S4 for parameters and symbols). The basic model described in the main text (eqs. 1-4) was extended by a second pathogen with deviating virulence (***v_2_***) and spore decay rate (*k*_2_). To investigate the invasibility of mutant pathogens, we treated its spore numbers (*W*_2_) and numbers of infected adults (*I*_2_) as additional variables. The presence or absence of a trade-off between spore production and virulence was modeled by *z*_2_ = *B*(*v*_2_+*d*)/(*v*_2_+*x*) and *z*_2_ = *B*(*v*_2_+*d*)*,* respectively. Note that the model does not allow double infections of the two pathogens. The full model consists of the following six differential equations.

$\frac{dA}{dt}=m\left( S+I+I_{2} \right)\left( 1-\frac{A}{C} \right)-uA,$ (5)

${\frac{dS}{dt}=-\beta S(W+W}_{2})+lA-(d+m)S,$ (6)

$\frac{dI}{dt}=\beta WS-(v+d+m)I,$ (7)

$\frac{dI_{2}}{dt}=\beta W_{2}S-(v_{2}+d+m)I_{2},$ (8)

$\frac{dW}{dt}=zI-kW,$ (9)

$\frac{dW_{2}}{dt}={z_{2}I}_{2}-k_{2}W_{2}.$ (10)

For the simulations, we used parameter values that were either measured in own experiments or taken from literature (see table S4 for details). Observed virulence levels where estimated by simulating the survival assay. All simulations started with susceptible larvae and spores in the environment only. We used the following initial conditions: ***A***(0)=0, ***S***(0)=100, ***I***(0)=0, ***I***_2_(0)=0, ***W***(0)=1000.

To investigate whether increased virulence can lead to host extinction, we first conducted a standard fixpoint analysis for model (1)-(4). Four equilibria where found, two out of which were stable. The first stable equilibrium corresponds to the complete absence of spores and hosts. The second stable equilibrium in relation to virulence is detailed in Figure S4. We then investigated virulence evolution in the full model (5)-(10). For this, the fitness of a rare mutant (r) with deviating virulence was calculated as the leading eigenvalue of the Jacobian matrix in disease free state. It computed to

$$r=\frac{1}{2z}\left( -z\left( d+k2+m+v2 \right)\sqrt{z(z\left( d+k2+m+v2 \right)^{2}+4(d\left( -k2z+k1z2 \right)+k1z2\left( m+v \right)-k2z(m+v2)} \right)$$

**Table S1.** Start and end *P. whitei* concentrations of evolved populations.

| **Starting Concentration** | **Replicate** | **End Concentration** |
| --- | --- | --- |
| 10^2^(low) | 1 | 3.7X10^6^ |
|  | 2 | 5X10^6^ |
|  | 3 | 2.2X10^6^ |
|  | 5 | 4.5X10^6^ |
|  | 7 | 5.6X10^6^ |
|  |  |  |
| 10^3^(intermediate) | 2 | 2.5X10^5^ |
|  | 3 | 2.3X10^6^ |
|  | 4 | 1.4X10^6^ |
|  | 5 | 3X10^6^ |
|  | 6 | 2.1X10^6^ |
|  | 7 | 6.1X10^6^ |
|  |  |  |
| 10^4^(high) | 2 | 1.6X10^6^ |
|  | 3 | 3.7X10^6^ |
|  | 4 | 2.4X10^6^ |
|  | 5 | 3.3X10^6^ |
|  | 6 | 2.6X10^6^ |
|  | 7 | 4.6X10^6^ |

Table **S2**. p values of pairwise ordinal-log-rank tests comparing survival curves among a tested *P. whitei* isolates. Multiple testing was accounted for using the “fdr” correction.

| Microsporidia isolate | Ancestral | Intermediate, 1 | Intermediate, 2 | Intermediate, 4 | Intermediate, 5 | Intermediate, 7 | Low, 3 | Low, 4 | Low, 5 | Low, 6 |
| --- | --- | --- | --- | --- | --- | --- | --- | --- | --- | --- |
| Ancestral | 0.7 | - | - | - | - | - | - | - | - | - |
| Intermediate, 1 | 0.8 | 0.9 | - | - | - | - | - | - | - | - |
| Intermediate, 2 | 0.3 | 0.1 | 0.1 | - | - | - | - | - | - | - |
| Intermediate, 4 | 0.5 | 0.6 | 0.6 | 0.1 | - | - | - | - | - | - |
| Intermediate, 5 | 0.5 | 0.3 | 0.4 | 0.6 | 0.1 | - | - | - | - | - |
| Intermediate, 7 | 0.6 | 0.4 | 0.5 | 0.5 | 0.2 | 0.8 | - | - | - | - |
| Low, 3 | 0.9 | 0.6 | 0.7 | 0.4 | 0.4 | 0.6 | 0.7 | - | - | - |
| Low, 4 | 0.5 | 0.3 | 0.4 | 0.5 | 0.1 | 0.9 | 0.9 | 0.6 | - | - |
| Low, 5 | 0.4 | 0.1 | 0.3 | 0.9 | 0.1 | 0.7 | 0.6 | 0.5 | 0.6 | - |
| Low, 6 | 1.0 | 0.7 | 0.8 | 0.3 | 0.5 | 0.5 | 0.7 | 0.9 | 0.5 | 0.4 |

**Table S3.** Results of binomial tests comparing proportions of alive and dead beetles following exposure to each of the evolved P. whitei isolates in comparision to the ancestral isolate. In all cases comparison is to the proportion of indiviuals alive in the ancestral treatment on eac day.

| Days | Treatment | Replicate | Successes | Trials | p value |
| --- | --- | --- | --- | --- | --- |
| 6, 8, 10 | Intermediate | 1 | 29 | 29 | n/a |
| and 12 |  | 2 | 25 | 25 | n/a |
|  |  | 4 | 26 | 26 | n/a |
|  |  | 5 | 25 | 25 | n/a |
|  |  | 7 | 27 | 27 | n/a |
|  | Low | 3 | 27 | 27 | n/a |
|  |  | 4 | 26 | 26 | n/a |
|  |  | 5 | 29 | 29 | n/a |
|  |  | 6 | 29 | 29 | n/a |
|  |  | 7 | 27 | 27 | n/a |

| 16 | Intermediate | 1 | 29 | 29 | 0.0677 |
| --- | --- | --- | --- | --- | --- |
|  |  | 2 | 25 | 25 | 0.1036 |
|  |  | 5 | 25 | 25 | 0.1036 |
|  |  | 4 | 26 | 26 | 0.1056 |
|  |  | 7 | 26 | 26 | 0.1056 |
|  | Low | 6 | 28 | 28 | 0.06714 |
|  |  | 5 | 29 | 29 | 0.0677 |
|  |  | 3 | 26 | 26 | 0.1056 |
|  |  | 7 | 26 | 26 | 0.1056 |
|  |  | 4 | 25 | 25 | 0.5182 |
| 18 | Intermediate | 2 | 23 | 25 | 0.295 |
|  |  | 1 | 29 | 29 | **0.005976** |
|  |  | 7 | 26 | 26 | **0.009223** |
|  |  | 5 | 25 | 25 | **0.01516** |
|  |  | 4 | 25 | 26 | 0.07107 |
|  | Low | 7 | 22 | 26 | 1 |
|  |  | 5 | 29 | 29 | **0.005976** |
|  |  | 6 | 27 | 28 | **0.04759** |
|  |  | 3 | 25 | 26 | 0.07107 |
|  |  | 4 | 24 | 26 | 0.3009 |
| 21 | Intermediate | 2 | 23 | 25 | 0.295 |
|  |  | 1 | 29 | 29 | **0.005976** |
|  |  | 5 | 25 | 25 | **0.01516** |
|  |  | 4 | 24 | 26 | 0.3009 |
|  |  | 7 | 24 | 26 | 0.3009 |
|  | Low | 7 | 22 | 26 | 1 |
|  |  | 5 | 28 | 29 | **0.0486** |
|  |  | 3 | 25 | 26 | 0.07107 |
|  |  | 4 | 23 | 26 | 0.6074 |
|  |  | 6 | 24 | 28 | 0.8065 |

| 28 | Intermediate | 4 | 20 | 26 | 1 |
| --- | --- | --- | --- | --- | --- |
|  |  | 2 | 23 | 25 | 0.06178 |
|  |  | 5 | 23 | 25 | 0.06178 |
|  |  | 1 | 25 | 29 | 0.2009 |
|  |  | 7 | 22 | 26 | 0.3648 |
|  | Low | 7 | 20 | 26 | 1 |
|  |  | 3 | 23 | 26 | 0.171 |
|  |  | 6 | 27 | 28 | 0.3852 |
|  |  | 5 | 23 | 28 | 0.5136 |
|  |  | 4 | 19 | 26 | 0.8218 |
| 30 | Intermediate | 7 | 20 | 26 | 1 |
|  |  | 5 | 23 | 25 | 0.06178 |
|  |  | 2 | 22 | 25 | 0.1675 |
|  |  | 1 | 25 | 29 | 0.2009 |
|  |  | 4 | 19 | 26 | 0.8218 |
|  | Low | 3 | 21 | 26 | 0.652 |
|  |  | 6 | 19 | 28 | 0.3852 |
|  |  | 4 | 18 | 26 | 0.4992 |
|  |  | 5 | 23 | 28 | 0.5136 |
|  |  | 7 | 19 | 26 | 0.8218 |
| 34 | Intermediate | 2 | 19 | 25 | 0.825 |
|  |  | 4 | 13 | 26 | **0.02704** |
|  |  | 5 | 21 | 25 | 0.1902 |
|  |  | 1 | 22 | 29 | 0.6854 |
|  |  | 7 | 18 | 26 | 0.8289 |
|  | Low | 6 | 17 | 28 | 0.2134 |
|  |  | 4 | 15 | 25 | 0.2663 |
|  |  | 3 | 16 | 26 | 0.2798 |
|  |  | 5 | 18 | 28 | 0.4063 |
|  |  | 7 | 17 | 26 | 0.5167 |

| 36 | Intermediate | 4 | 10 | 26 | **0.002499** |
| --- | --- | --- | --- | --- | --- |
|  |  | 5 | 21 | 25 | 0.09031 |
|  |  | 7 | 14 | 26 | 0.1424 |
|  |  | 1 | 22 | 29 | 0.43 |
|  |  | 2 | 16 | 25 | 0.6725 |
|  | Low | 6 | 12 | 28 | **0.00741** |
|  |  | 3 | 14 | 26 | 0.1424 |
|  |  | 7 | 15 | 26 | 0.2951 |
|  |  | 5 | 17 | 29 | 0.3208 |
|  |  | 4 | 15 | 25 | 0.3979 |
| 38 | Intermediate | 1 | 22 | 29 | 0.127 |
|  |  | 4 | 10 | 26 | **0.02607** |
|  |  | 5 | 20 | 25 | 0.0635 |
|  |  | 7 | 12 | 26 | 0.1593 |
|  |  | 2 | 16 | 25 | 0.8393 |
|  | Low | 4 | 14 | 25 | 0.684 |
|  |  | 6 | 9 | 28 | **0.002997** |
|  |  | 3 | 12 | 26 | 0.1593 |
|  |  | 7 | 15 | 26 | 0.8414 |
|  |  | 5 | 17 | 29 | 0.8504 |
| 40 | Intermediate | 4 | 9 | 26 | **0.008353** |
|  |  | 5 | 19 | 25 | 0.1516 |
|  |  | 7 | 12 | 26 | 0.1593 |
|  |  | 1 | 21 | 29 | 0.2543 |
|  |  | 2 | 16 | 25 | 0.8393 |
|  | Low | 4 | 14 | 25 | 0.684 |
|  |  | 6 | 9 | 28 | **0.002997** |
|  |  | 3 | 11 | 26 | 0.06955 |
|  |  | 5 | 16 | 29 | 0.5714 |
|  |  | 7 | 15 | 26 | 0.8414 |

| 42 | Intermediate | 4 | 8 | 26 | **0.002288** |
| --- | --- | --- | --- | --- | --- |
|  |  | 7 | 12 | 26 | 0.1593 |
|  |  | 5 | 18 | 25 | 0.3081 |
|  |  | 1 | 20 | 29 | 0.4486 |
|  |  | 2 | 16 | 25 | 0.8393 |
|  | Low | 4 | 14 | 25 | 0.684 |
|  |  | 6 | 9 | 28 | **0.002997** |
|  |  | 3 | 11 | 26 | 0.06955 |
|  |  | 5 | 14 | 29 | 0.1858 |
|  |  | 7 | 15 | 26 | 0.8414 |
| 44 | Intermediate | 2 | 15 | 25 | 1 |
|  |  | 4 | 7 | 26 | **0.000855** |
|  |  | 7 | 11 | 26 | 0.06955 |
|  |  | 5 | 18 | 25 | 0.3081 |
|  |  | 1 | 19 | 29 | 0.7052 |
|  | Low | 4 | 14 | 25 | 0.684 |
|  |  | 6 | 9 | 28 | **0.002997** |
|  |  | 3 | 11 | 26 | 0.06955 |
|  |  | 5 | 14 | 29 | 0.1858 |
|  |  | 7 | 15 | 26 | 0.8414 |
| 46 | Intermediate | 2 | 15 | 25 | 1 |
|  |  | 4 | 7 | 26 | **0.000855** |
|  |  | 7 | 11 | 26 | 0.06955 |
|  |  | 5 | 18 | 25 | 0.3081 |
|  |  | 1 | 19 | 29 | 0.7052 |
|  | Low | 4 | 14 | 25 | 0.684 |
|  |  | 6 | 9 | 28 | **0.002997** |
|  |  | 5 | 10 | 28 | **0.01051** |
|  |  | 3 | 11 | 26 | 0.06955 |
|  |  | 7 | 15 | 26 | 0.8414 |

| 48 | Intermediate | 1 | 18 | 29 | 1 |
| --- | --- | --- | --- | --- | --- |
|  |  | 2 | 15 | 25 | 1 |
|  |  | 4 | 7 | 26 | **0.000855** |
|  |  | 7 | 11 | 26 | 0.06955 |
|  |  | 5 | 18 | 25 | 0.3081 |
|  | Low | 4 | 14 | 25 | 0.684 |
|  |  | 6 | 9 | 28 | **0.002997** |
|  |  | 5 | 10 | 28 | **0.01051** |
|  |  | 3 | 11 | 26 | 0.06955 |
|  |  | 7 | 15 | 26 | 0.8414 |
| 51 | Intermediate | 1 | 18 | 29 | 1 |
|  |  | 2 | 15 | 25 | 1 |
|  |  | 4 | 7 | 26 | **0.000855** |
|  |  | 7 | 10 | 26 | **0.02607** |
|  |  | 5 | 18 | 25 | 0.3081 |
|  | Low | 4 | 14 | 25 | 0.684 |
|  |  | 6 | 9 | 28 | **0.002997** |
|  |  | 5 | 10 | 28 | **0.01051** |
|  |  | 3 | 11 | 26 | 0.06955 |
|  |  | 7 | 15 | 26 | 0.8414 |
| 53 | Intermediate | 1 | 18 | 29 | 1 |
|  |  | 2 | 15 | 25 | 1 |
|  |  | 4 | 7 | 26 | **0.000855** |
|  |  | 7 | 9 | 26 | **0.008353** |
|  |  | 5 | 18 | 25 | 0.3081 |
|  | Low | 4 | 14 | 25 | 0.684 |
|  |  | 6 | 9 | 28 | **0.002997** |
|  |  | 5 | 10 | 28 | **0.01051** |
|  |  | 3 | 11 | 26 | 0.06955 |
|  |  | 7 | 15 | 26 | 0.8414 |

**Table S4.** Parameter values included in the model. These parameters were used to run simulations for all figures.

| **Parameter** | **Description** | **Value** | **Reference** |
| --- | --- | --- | --- |
| **m** | Maturation constant | 0.03 | Calculated from life tables in (Sokoloff, 1972) |
| **β** | transmission constant | 0.00083 |  |
| **k** | propagule decay constant | 0.0075 | Calculated from spore longevity values (Milner, 1972) |
| **u** | adult removal constant | 0.07 | Based on artificial removal of adults in our experimental conditions. Under standard laboratory conditions u would take a value of 0.003 |
| **C** | Carrying capacity | 100 | Imposed by the experiment |
| **l** | birth of susceptible larvae | 0.33 | Rafaluk (Unpublished) |
| **d** | background larval mortality | 0.03 | Calculated from life tables in (Sokoloff, 1972) |
| **v** | Pathogen induced mortality: virulence | 0.03,  for experimental estimates see S3. | Disease induced mortality, calculated from average time point of death |
| **B** | Maximum number of propagules shed into the environment shed upon host death | 750000 | Calculated based on spore counts (Rafaluk unpublished) |
| **z** | z Spore production upon host death. A | Without trade-off:z=B(v+d)  With trade-off:  z=B(v+d)/(v+x) | Trade-off assumptions following Bonhoeffer *et al.*, 1996 |
| **x** | trade-off constant | 0.15 | Trade-off assumptions following Bonhoeffer *et al.*, 1996. At 0.15 the wild-type virulence is evolutionary stable unless spore mortality is decreased. |
|  |  |  |  |

**Figure S1.** Diagram of experimental set up of the evolution experiment.


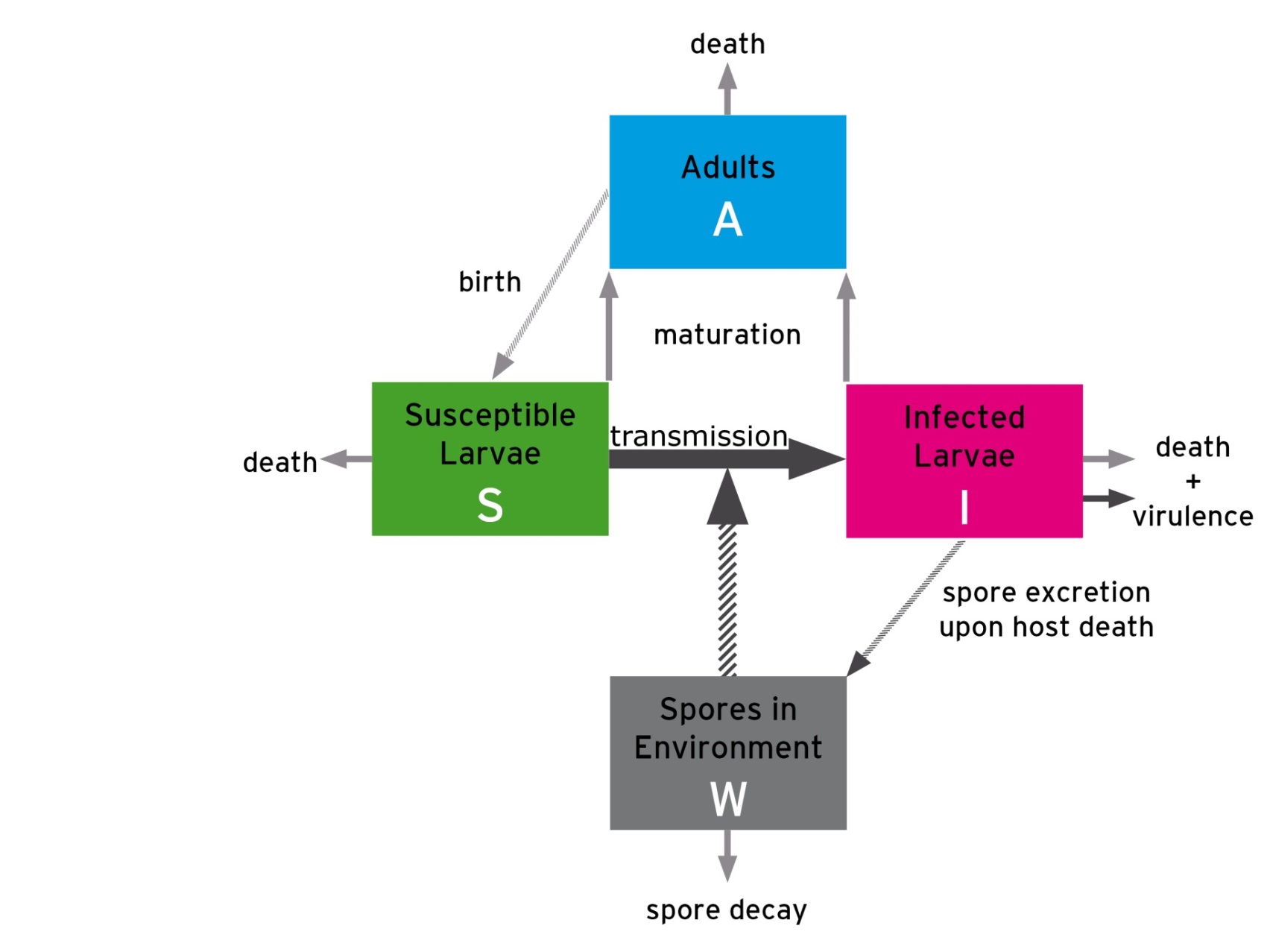


**Figure S2.** Schematic representation of the epidemiological model.


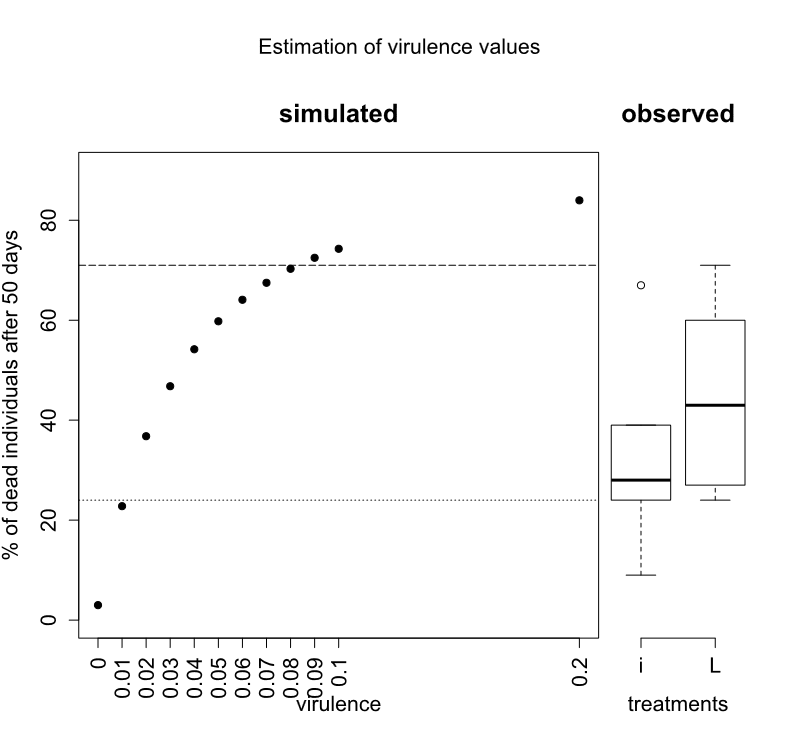


Figure S3. Here we show how we estimated virulence levels from the survival assay. The boxplots on the left indicate the proportion of dead that are alive after 50 days at the end of the survival assay. Treatment i represents parasites isolated from the intermediate experimental condition and, treatment L from the low experimental condition. The black dots on the left show the simulated valuesvalues on the left show simulated proportions of dead individuals under survival assay conditions. The dashed line indicates the highest observed host mortality. The dotted line indicates average mortality caused by wild parasites before evolution. Survival was simulated from the model in the absence of reproduction with differing levels of virulence. The following parameter values were used in this simulation propagule transmission (b) = 0.00083; Larval maturation (m) = 0.03; Adult removal (u) = 0.003; Carrying capacity (C) = 100; Larval background mortality (d) = 0.03; Spore mortality (k) = 0.0075; Spore shedding (B) = 750000 and (B2) = 750000.

Figure S4. Here we show stable equilibrium points (y-axis) calculated from the model A, with just one genotype, over likely levels of virulence under experimental conditions (ranging between 0.01 and 0.09, see figure S3). The following parameter values were used in this simulation propagule transmission (b) = 0.00083; Larval maturation (m) = 0.03; Adult removal (u) = 0.07; Carrying capacity (C) = 100; Larval birth (l) = 0.33; Larval background mortality (d) = 0.03; Spore mortality (k) = 0.0075; Spore shedding (B) = 750000. As described above, in our experimental setup adult removal was higher than in standard laboratory conditions. Our simulations suggest that this decrease in adult lifespan in combination with virulence evolution caused low host densities. For high levels of virulence host densities never become negative but are maintained well below one individual in the absence of migration this is virtually extinction. For the here tested parameter values parasites do not go extinct unless hosts are completely absent.

References

Anderson, R.M., May, R.M. (1981) The Population Dynamics of Microparasites and Their Invertebrate Hosts. *Philosophical Transactions of the Royal Society of London. B, Biological Sciences*. **291**(1054), 451–524.

Bonhoeffer, S., Lenski, R.E., Ebert, D. (1996) The Curse of the Pharaoh: The Evolution of Virulence in Pathogens with Long Living Propagules. *Proceedings of the Royal Society B: Biological Sciences*. **263**(1371), 715–721.

Milner, R.J. (1972) *Nosema whitei*, a microsporidan pathogen of some species of *Tribolium*: I. Morphology, life cycle, and generation time. *Journal of Invertebrate Pathology*. **19**(2), 231–238.

Sokoloff, A. (1972) *The biology of Tribolium*. Gloucestershire, Clarendon Press.
